# Validation of Acute Myocardial Infarction Cases in the National Health Insurance Research Database in Taiwan

**DOI:** 10.2188/jea.JE20140076

**Published:** 2014-11-05

**Authors:** Ching-Lan Cheng, Cheng-Han Lee, Po-Sheng Chen, Yi-Heng Li, Swu-Jane Lin, Yea-Huei Kao Yang

**Affiliations:** 1 Institute of Clinical Pharmacy and Pharmaceutical Sciences, College of Medicine, National Cheng Kung University, Tainan, Taiwan; 2 Health Outcome Research Center, National Cheng Kung University, Tainan, Taiwan; 3 Department of Internal Medicine, National Cheng Kung University Hospital, Tainan, Taiwan; 4 University of Illinois at Chicago, Chicago, IL, USA

**Keywords:** acute myocardial infarction, NHIRD, Taiwan, validity, pharmacoepidemiology

## Abstract

**Background:**

The aim of this study was to determine the validity of acute myocardial infarction (AMI) diagnosis coding in the National Health Insurance Research Database (NHIRD) by cross-comparisons of discharge diagnoses listed in the NHIRD with those in the medical records obtained from a medical center in Taiwan.

**Methods:**

This was a cross-sectional study comparing records in the NHIRD and discharge notes in one medical center (DNMC) in the year 2008. Positive predictive values (PPVs) for AMI diagnoses were evaluated by reviewing the relevant clinical and laboratory data recorded in the discharge notes of the medical center. Agreement in comorbidities, cardiac procedures, and antiplatelet agent (aspirin or clopidogrel) prescriptions between the two databases was evaluated.

**Results:**

We matched 341 cases of AMI hospitalizations from the two databases, and 338 cases underwent complete chart review. Of these 338 AMI cases, 297 were confirmed with clinical and lab data, which yielded a PPV of 0.88. The consistency rate for coronary intervention, stenting, and antiplatelet prescription at admission was high, yielding a PPV over 0.90. The percentage of consistency in comorbidity diagnoses was 95.9% (324/338) among matched AMI cases.

**Conclusions:**

The NHIRD appears to be a valid resource for population research in cardiovascular diseases.

## INTRODUCTION

The National Health Insurance Research Database (NHIRD) is a population-based database derived from the claims data of the National Health Insurance (NHI) program, which is a mandatory enrollment and single payment system created in 1995 that covers over 99% of Taiwan’s population and provides opportunities to conduct longitudinal studies to evaluate treatment outcomes and drug-disease associations.^1^ However, for a database to be useful for research, its data must be valid.

Diagnosis data in medical data sets are essential in studies of health care research and are often used to estimate disease incidence/prevalence, assess health outcomes, adjust for risks, and evaluate health-system performance and policy intervention.^2^ In Taiwan, the NHIRD has been widely used in epidemiological studies of cardiovascular disease (CVD) or drug-related cerebrovascular disorders.^3^^,^^4^ The epidemiology of CVD is of special interest to researchers in the Asia-Pacific region because of the notably higher prevalence of stroke there than in Western countries.^5^^,^^6^ Our previous study documented the high accuracy (97.85%) of the admission diagnoses of ischemic stroke in the NHIRD, thus deeming the database appropriate for research in this disease area.^7^

The Westernization of lifestyles, including the increase in fat consumption, has been linked to the prevalence of obesity and/or diabetes and may have increased incidence of acute myocardial infarction (AMI) in Asian countries.^8^ Access to findings from studies on epidemiological features of AMI, the treatment strategy associated with the health outcome in AMI cases, and drug-related AMI in Asian populations has increased recently.^9^^–^^11^ However, details regarding the validation of AMI diagnosis and the recording of AMI in electronic databases that are commonly used in research have not been reported.

Thus, the first objective of this study was to assess the accuracy of diagnosis by reviewing the medical charts of patients with AMI, and the second objective was to evaluate the agreement of procedure, comorbidities, and prescriptions of antiplatelet agents (aspirin or clopidogrel) in these cases between the medical charts and the claims database.

## METHODS

### Data sources and record linkage

Data from the NHIRD and the discharge notes of medical records from a tertiary medical center (DNMC) in Southern Taiwan for 2008 were obtained to validate the corresponding information in the NHIRD. This medical center is a health care center with approximately 1200 beds and an occupancy rate of about 78%. The discharge notes of each patient included the following information: chief complaints, present illness, treatment procedures, and all diagnoses made during their hospitalization.

The NHIRD is derived from the registration files and original claims data of the NHI program and is maintained by the National Health Research Institutes. The data elements available for each hospital admission in the NHIRD includes patient sex, date of birth, date of admission, diagnosis at discharge (up to 5 diagnoses), procedures undergone (up to 5), expenditures, and detailed prescriptions.

Information in the NHIRD that could be used to identify individual patients and healthcare providers is scrambled to protect patient privacy and confidentiality.^12^ Being unable to directly match the NHIRD data to medical records by patient-specific identifiers, previous studies have proposed alternative methods of identifying specific medical records without patient identifiers.^13^^–^^15^ One method involves using probabilistic record linkage that simultaneously matches multiple non-unique characteristics of patients, such as name initials, date of birth, and gender, to identify records of the same individual from different data sources.^16^

First, we extracted from both the NHIRD and the DNMC the records of patients who were admitted to the medical center in 2008 for AMI (ICD-9-CM code: 410xx). Afterward, date of birth, gender, admission date, and discharge date were used to match records from the two extracted data files.

Two types of cases were excluded from the study because of the inability to accurately link records across the two databases. One type of exclusion was used when there were multiple patients who shared the same attributes in the four matching variables. Due to the large number of records in the NHIRD and DNMC databases, it was possible that more than one patient might share the same date of birth, gender, admission date, and discharge date. The second exclusion type involved any records with missing values in any of the four matching variables. Further, because medical records are considered the gold standard in validation studies, only those records that could be matched to the medical records of the DNMC were included for analysis. If a patient had multiple hospitalizations for AMI, only the first episode was included in the study.

### Validating diagnoses of AMI

To ensure the accuracy of recorded diagnoses, we further reviewed the medical charts to validate diagnoses of AMI and type of AMI (ST elevation or non-ST elevation) for the matched cases in the DNMC. From DNMC information, one author abstracted hospital medical records using a structured chart abstraction program to comply with the diagnosis criteria of AMI established by the World Health Organization (WHO). Abstracted data from the patient charts were entered directly into an electronic database. Data elements included detailed information relevant to the diagnosis of AMI, such as patients’ chief complaints at admission (eg, chest pain), electrocardiography (ECG) data, laboratory data (eg, creatine-kinase [CK], creatine-kinase MB fraction [CK-MB], or troponin-T [TnT]), procedures (eg, percutaneous coronary intervention [PCI], coronary artery bypass grafting [CABG], or stenting), and prescriptions of antiplatelet agents (eg, aspirin or clopidogrel) during admission and at the first outpatient visit after discharge.

All charts were independently reviewed by two cardiologists. When there was disagreement between the two reviewers, consensus was sought through discussion with a third expert. We also evaluated the data quality of patient charts with WHO criteria; that is, at least two of the following three required conditions must be documented in a medical chart to confirm an AMI diagnosis: chest discomfort characteristic of ischemia, ECG changes indicative of ischemia (ST elevation/Q waves or ST depression), and elevations in serum markers typical of myocardial injury (CK-MB, TnT). ST elevation myocardial infarction (STEMI, ICD9 codes: 410.0–410.6) and non-ST elevation myocardial infarction (NSTEMI, ICD9 codes: 410.7 or 410.9) were distinguished based on ECG findings.^17^^–^^19^

### Validating procedure and prescriptions of antiplatelet agents

In addition to verifying the diagnosis, we also validated the accuracy in documenting procedures of coronary interventions between the DNMC and the NHIRD and analyzed the consistency rate between the two databases in aspirin and clopidogrel prescriptions during hospitalization and at the first outpatient visit within one year after discharge. During the evaluations, procedures and prescriptions of antiplatelet agents recorded in the DNMC were used as the gold standard for comparisons.

### Agreement in discharge diagnosis

We determined the consistency of all discharge diagnoses between the DNMC and the NHIRD records, not only whether the matched cases had the same diagnosis codes but also whether the codes appeared in the same order. For example, if the diagnoses on a NDMC record were listed as 410.91, 250.00, and 401.9 but the matched record from the NHIRD showed 410.91, 401.9, and 250.00, the coding would be considered as inconsistent because the order of the diagnoses was different between the two data sources.

### Statistical Analysis

The matching rate was presented as the number of matched cases divided by the number of cases retrieved from the DNMC (the gold standard). The validity of using the ICD-9 410.xx code to identify matched cases of AMI was assessed by calculating the positive predictive value (PPV) using medical records (of confirmed cases after review by the cardiologists) as the gold standard. The agreement rate between the two reviewers was calculated using the agreement cases divided by the total cases. In addition, we estimated the PPV of principal diagnosis, antiplatelet therapy, and cardiac procedures of confirmed AMI cases. Further, different criteria were used to evaluate sensitivity and PPV of the diagnosis code of AMI in the NHIRD, such as “principal diagnosis with antiplatelet” or “principal diagnosis with percutaneous transluminal coronary angioplasty (PTCA)”.

To ensure validity of procedures and aspirin/clopidogrel exposure, we defined sensitivity as the probability that the procedure/antiplatelet agents recorded in the medical chart (denominator) by a doctor were also recorded in the NHIRD (numerator). PPV is the conditional probability that claims of procedures/antiplatelet agents in the NHIRD (denominator) were actually present in the DNMC records (numerator). For agreement among discharge diagnoses for each AMI hospitalization, percentage of consistency between the two databases was calculated for linkage cases.

All computations and 95% confidence intervals (CIs) for binominal proportions were performed with SAS version 9.2 (SAS Institute Inc, Cary, NC, USA). This study was reviewed and approved by the Institutional Review Board of the National Cheng Kung University Medical Center (ER-95-137).

### Role of the Funding Source

This research was funded by the National Cheng Kung University Hospital (NCKU-10 101 002). The funding source had no role in the design, analysis, interpretation, or reporting of results or in the decision to submit the manuscript for publication.

## RESULTS

### Selection of population

**Figure 1.**
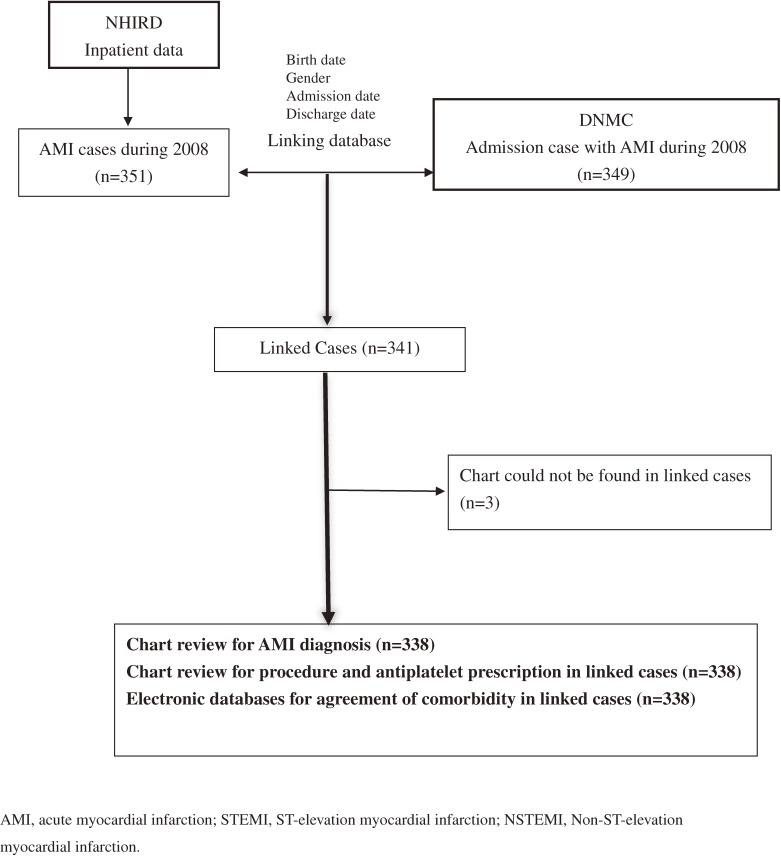
Flow chart of study procedure

We extracted a total of 349 AMI cases from the DNMC and 351 AMI cases from the NHIRD. Linkage was achieved in 341 AMI cases, with a linkage rate of 97% (341/351). Three cases were excluded due to misplacement of medical charts. A total of 338 cases completed the chart review (Figure 1). The mean patient age was 68.1 years (standard deviation 13), and 71% were male. Among the linked cases, 83% (281/338) had AMI listed as the principal diagnosis in the NHIRD, and 280 of the 281 cases also had AMI listed at the same position (principal diagnosis) in the matched DNMC records.

### Validation of AMI

**Figure 2.**
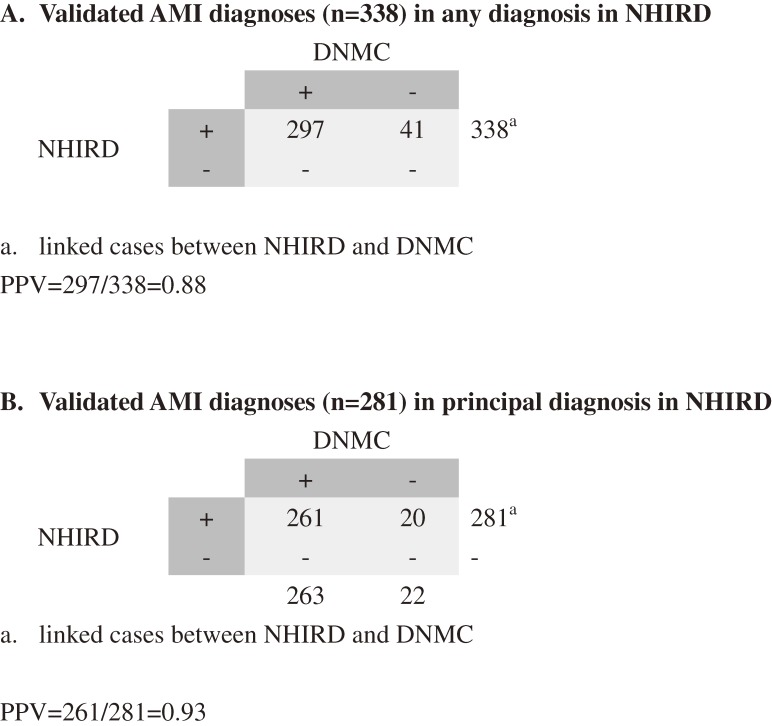
Validation of AMI diagnoses in any position or principal position

Based on medical records and laboratory data from the DNMC, the AMI diagnoses (regardless of the order the diagnosis appeared) of 297 cases were confirmed, yielding a PPV of 0.88 (Figure 2A). The PPV increased to 0.93 (261/281) when using only the principal diagnosis in the NHIRD (Figure 2B). Among the 297 confirmed cases, 202 (68%) had complaints of chest pain, 288 (97%) were found to have ECG changes indicating ischemia, and 279 (94%) presented with typical elevations in serum markers of myocardial injury (CK-MB or TnT). In addition, 146 of the 297 patients were STEMI, 147 were NSTEMI, and 4 were unspecified. In addition, 90.4% of the STEMI cases had accurate codes of “410.0–410.6” and 97.9% of the NSTEMI cases had accurate codes of “410.7 and 410.9”.

The two reviewers agreed on the diagnosis in 84% of cases, including 258 cases confirmed with AMI diagnosis and 27 cases determined as not being admitted due to AMI. Among the linked cases, criteria with principal diagnosis of AMI in the NHIRD presented a high PPV, and any diagnosis of AMI with at least one antiplatelet agent presented a high sensitivity in the NHIRD (Table 1).

**Table 1.** Estimated sensitivity and PPV in the NHIRD using different criteria (*n* = 338)^a^

Criteria	NHIRD	DNMC	Sensitivity^c^	PPV^d^
Any diagnosis “410” + antiplatelet^b^	338	284	0.96	0.84
Any diagnosis “410” + heparin + antiplatelet	274	256	0.86	0.93
Principal diagnosis “410”	281	261	0.88	0.92
Principal diagnosis “410” + antiplatelet	274	254	0.86	0.93
Principal diagnosis “410” + heparin + antiplatelet	241	230	0.77	0.95
Principal diagnosis “410” + Catheterization	224	212	0.71	0.95
Principal diagnosis “410” + PTCA	194	184	0.62	0.95

DNMC, discharge notes of medical records from a tertiary medical center; NHIRD, National Health Insurance Research Database; PPV, positive predictive value; PTCA, percutaneous transluminal coronary angioplasty.

^a^Linked cases between the NHIRD and DNMC; 338 cases complete chart review; 297 cases with confirmed AMI in the DNMC were considered the gold standard.

^b^Antiplatelets, including aspirin, clopidogrel, dipyridamole, and ticlopidine.

^c^Patients who received a diagnosis (and prescription) in the DNMC and were also present in the NHIRD. For example, the sensitivity for any (principal or secondary) diagnosis of “410” + antiplatelet was 0.96 (284/297).

^d^The proportion of records in the NHIRD that could also be found in the DNMC with certain criteria; for example, 284 cases in the DNMC with any diagnosis “410” + antiplatelet were present in NHIRD cases, which resulted in a PPV of 0.84 (284/338).

### Validation of procedure and prescriptions of antiplatelet agents

In the reviewed cases from the DNMC, the sensitivity and PPV was more than 0.9 for catheterization, PCI, CABG, and stenting (Table 2). There was also a high PPV for the prescription of antiplatelet agents (aspirin/clopidogrel) during hospitalization for MI.

**Table 2.** Validation of procedure and prescription of aspirin and clopidogrel (*n* = 338)^a^

	DNMC (+)	DNMC (−)	DNMC (+)	DNMC (−)	Sensitivity^b^ (95% CI)	Specificity^c^ (95% CI)	PPV^d^ (95% CI)	NPV^e^ (95% CI)

	NHIRD (+)	NHIRD (+)	NHIRD (−)	NHIRD (−)				
**Procedure/intervention**								
Catheterization	240	3	8	87	0.97 (0.94–0.99)	0.97 (0.91–0.99)	0.99 (0.96–0.99)	0.91 (0.84–0.96)
PTCA	205	5	6	122	0.97 (0.94–0.99)	0.96 (0.91–0.99)	0.98 (0.94–0.99)	0.95 (0.90–0.98)
CABG	22	2	2	312	0.92 (0.73–0.99)	0.99 (0.98–0.99)	0.92 (0.73–0.99)	0.99 (0.98–0.99)
Stenting	184	1	19	134	0.90 (0.86–0.94)	0.99 (0.96–0.99)	0.99 (0.97–0.99)	0.88 (0.81–0.92)

**Prescription at admission**								
Aspirin	293	7	9	29	0.97 (0.95–0.99)	0.80 (0.64–0.92)	0.98 (0.95–0.99)	0.76 (0.60–0.88)
Clopidogrel	299	4	4	31	0.98 (0.96–0.99)	0.88 (0.73–0.97)	0.98 (0.97–0.99)	0.88 (0.73–0.97)

**Prescription at first visit after discharge within one year**								
Aspirin	199	34	4	100	0.98 (0.95–0.99)	0.75 (0.66–0.82)	0.85 (0.80–0.90)	0.96 (0.90–0.99)

Clopidogrel	205	38	4	91	0.98 (0.95–0.99)	0.70 (0.62–0.78)	0.84 (0.79–0.88)	0.96 (0.89–0.99)

CABG, coronary artery bypass graft; DNMC, discharge notes of medical records from a tertiary medical center; NHIRD, National Health Insurance Research Database; NPV, negative predictive value; PPV, positive predictive value; PTCA, percutaneous transluminal coronary angioplasty.

^a^Linked cases between the NHIRD and DNMC; records in the DNMC were considered the gold standard.

^b^Proportion of patients who underwent a procedure (or received a prescription) in the DNMC and were also coded as such in the NHIRD; for example, the sensitivity of catheterization was 0.97 [240/(240 + 8)].

^c^Proportion of patients without a procedure record (or prescription) in the DNMC or in the NHIRD, such as specificity of catheterization = 87/(87 + 3) = 0.97.

^d^Proportion of records present in the NHIRD and confirmed to have a procedure record (or prescription) in the DNMC, such as PPV of catheterization = 240/(240 + 3)= 0.99.

^e^Proportion of records absent in the NHIRD and confirmed to not have a procedure record (or prescription) in the DNMC, such as NPV of catheterization = 87/(87 + 8) = 0.91.

### Agreement of five discharge diagnoses

The consistency in five discharge diagnoses of each AMI hospitalization (including those with ICD-9 410.xx) was 95.9% (324/338) among all linked AMI cases. There were 14 cases that had different discharge diagnoses between the two databases; of these, 5 had diagnoses in the DNMC that were not found in the NHIRD (case 1–case 5 in Table 3). One case (case 6) had myocardial infarction (MI) on the anteroseptal wall (410.12) listed in the DNMC record but on the anteroapical wall (410.11) in the NHIRD. Two cases (cases 13 and 14) had a history of malignancy but were coded differently in the two databases.

**Table 3.** Details regarding inconsistencies in discharge diagnosis codes between two datasets

Case no.	NHIRD diagnoses					DNMC diagnoses				
	
	1	2	3	4	5	1	2	3	4	5
1	410.11	414.01	250.00			410.11	414.01	250.00	**272.4**	
2	410.11	414.01	V02.61	401.9		410.11	414.01	**496**	401.9	V02.61
3	414.01	410.92	401.9			414.01	410.92	401.9	7**80.2**	
4	414.01	410.72	585	250.00		414.01	410.72	**584.9**	250.00	585
5	410.61	414.01	424.0			410.61	414.01	424.0	**413.9**	
6	**410.11**	428.0	584.9	790.7	427.31	428.0	**410.12**	584.9	790.7	427.31
7	410.41	424.0	**401.9**	272.4	250.00	410.41	424.0	**414.01**	272.4	250.00
8	410.41	414.01	511.9	**785.51**	427.31	410.41	414.01	511.9	**532.90**	427.31
9	410.71	414.01	411.1	**518.81**	532.40	410.71	414.01	411.1	**250.00**	532.40
10	410.71	414.01	578.9	280.0	**426.13**	410.71	414.01	578.9	280.0	**290.0**
11	414.01	410.41	428.0	584.9	**250.00**	410.41	414.01	428.0	584.9	**403.91**
12	414.01	410.72	250.00	496	**600.0**	414.01	**584.9**	410.72	250.00	496
13	410.41	414.01	427.1	434.91	**145.9**	410.41	414.01	427.1	434.91	**V10.22**
14	410.71	414.01	**151.9**	250.00	272.4	410.71	414.01	**V10.04**	250.00	272.4

DNMC, discharge notes of medical records from a tertiary medical center; NHIRD, National Health Insurance Research Database.

145.9: malignant neoplasm of unspecified parts of mouth

151.9: malignant, stomach, unspecified

250.00: diabetes

272.4: other and unspecified hyperlipidemia

290.0: senile dementia, uncomplicated

401.9: essential hypertension

403.91: hypertensive, chronic kidney disease

410.11: infarction: anteroapical

410.12: infarction: anteroseptal

413.9: other and unspecified angina pectoris

414.01: coronary atherosclerosis of native coronary artery

426.13: other second-degree atrioventricular block

496: chronic airway obstruction, not elsewhere classified

518.81: acute respiratory failure

532.90: Duodenal ulcer, unspecified

584.9: acute renal failure, unspecified

600.0: hypertrophy of prostate

780.2: syncope and collapse

785.51: cardiogenic shock

V10.22: personal history of malignant, larynx

## DISCUSSION

In the first stage of this study, we used medical records to confirm the diagnosis of AMI in discharge notes. Our findings suggested reliable diagnosing of AMI in this medical center, with PPVs of 0.88 (if based on principal or secondary diagnosis; Figure 2A) or 0.93 (if based only on principal diagnosis; Figure 2B), similar to previous AMI validation studies that also reported high PPV in principal diagnosis. Petersen et al confirmed the diagnosis of AMI in the administrative database of 96.9% of patients who were admitted for that diagnosis in the hospitals of the Department of Veterans Affairs in the United States.^20^ The PPV (the proportion of patients with 410.xx code for AMI who have been correctly diagnosed) was found to be 0.96 in the principal discharge diagnosis of the hospital discharge databases among patients who had MI in Canada.^21^ The accuracy of diagnosis coding was 0.94 for AMI from Medicare administrative databases when the coding was specified in the principal position.^22^ Recently, an FDA-funded project in the United States, the Mini-Sentinel pilot program, validated 153 potential cases of AMI with primary diagnoses of 410.0x and 410.1x from four health plans. The overall PPVs were 0.86, ranging from 0.76 to 0.94 across the four data partners.^23^

As it is impossible to review all cases in the administrative claims database, we estimated the sensitivity and PPV using different criteria based upon practice guidelines.^24^ As expected, the highest PPV was achieved in patients who had a primary diagnosis of “410” and either a PCI procedure or combined heparin and antiplatelet treatment (Table 1). Primary PCI should be performed within 12 hours for patients with ischemic symptoms, antiplatelet agents should be given after PCI, and heparin should be administered as needed to maintain a therapeutic active clotting time for patients undergoing primary PCI, according to practice guidelines (level of evidence: A).^24^^,^^25^ Because PCI, antiplatelet agents, and heparin are important in validating AMI cases, the sensitivity of identifying AMI cases in the NHIRD could be reduced if some patients in the databases did not receive primary PCI or medications due to contraindications.

Two types of AMI (STEMI and NSTEMI) are characterized by a typical rise and/or fall in the biomarkers of myocyte injury.^19^ The treatment strategy of practice guidelines is similar in the two groups, such as early PCI, initial anticoagulant therapy, and secondary prevention medications.^24^^,^^26^ However, some characteristic differences exist between patients with STEMI and NSTEMI, such as a higher short-term mortality in STEMI patients than NSTEMI patients and greater prevalence of comorbidities among patients with NSTEMI than STEMI.^27^ Thus, it is necessary to distinguish these two populations in controlled clinical trials or real-world practice.^28^ In the present study, we found that half of the confirmed AMI cases had STEMI, and more than 90% had correct ICD-9 coding; while among the NSTEMI cases, the correct ICD-9 coding rate was nearly 98%.

Most of the procedures recorded in the NHIRD could also be found in the charts of the DNMC. (Table 2) One possible reason that nearly 5% of patients with stenting could not be found in the NHIRD may be that some patients paid out of their pockets for the procedure due to the fact that drug-eluting stents were not covered by NHI before 2007. More than 95% of patients (284/297) with confirmed AMI in the current study had received at least one antiplatelet agent at admission. We found that not all of the aspirin prescriptions in the NHIRD were recorded in the DNMC and that the specificity and PPV were lower in ambulatory care than in inpatient prescriptions. We speculate that some patients may have transferred out of the DNMC in this study to other hospitals or clinics, and the follow-up data were not captured in the charts of the DNMC or the NHIRD records pertaining to the DNMC. While aspirin is likely to be an over-the-counter medication in Western countries, it is covered by the NHI program in Taiwan for specific health conditions and healthcare settings. It has been estimated that most aspirin prescriptions in outpatient settings were included in the NHIRD,^29^ which makes it very useful in conducting pharmacoepidemiological studies to investigate aspirin-related healthcare issues.

NHI allowed up to five diagnosis codes in admission cases for reimbursement. It is expected that the codes contain a primary diagnosis and four other diagnosis codes representing comorbidities during a hospitalization. We evaluated all diagnosis codes that were consistently positioned in the records of both databases (Table 3). There were only 5 cases that had different diagnosis codes (cases 8, 9, 10, 11, and 12); thus, our study confirmed the high accuracy of diagnosis coding among the matched cases, which supports the use of the NHIRD in estimating comorbidities in research. The primary reason for inconsistent coding could be due to data error, and it may occur at any step during data collection or data transfer from healthcare facilities to the NHIRD.^30^

### Limitations

Limitations should be noted in this study. First, results were limited to one medical center during a one-year study period. How the performance varies with time or with other provider systems needs further assessment in the future. However, since all the hospitals in Taiwan must be accredited to be eligible to contract with the NHI program and the data processing systems for reimbursement are the same among health care providers of the same level, it is likely that our results can be generalized to the other 27 NHI medical centers in Taiwan. Second, we were unable to evaluate the extent of data error (such as transfer data error, which may result from administrative procedures or data encryption in the NHIRD). Third, the study was carried out with 2008 data; the results might therefore not be applicable to more recent data. However, since there has been no significant change in ICD-9 codes, diagnostic criteria of AMI, or the administrative process in the NHIRD or DNMC since 2008, it is unlikely that the data quality would have changed significantly in more recent years. Finally, although there was high consistency in all diagnosis codes between the two databases, the current study only evaluated the validity of AMI (410.xx) recorded in medical charts. Further study is needed to evaluate the accuracy of accompanying comorbidity diagnoses among the AMI cases.

### Conclusions

Our study shows that PPV was 0.88 for the admission cases with AMI and increased to greater than 0.90 when using principal diagnosis with coronary intervention or antiplatelet agents in the NHIRD. The misclassification of STEMI or NSTEMI is minimal in the NHIRD. The agreement between the NHIRD and the DNMC was high in cardiac procedures, comorbidity diagnosis, and antiplatelet prescriptions. Overall, the NHIRD has a high accuracy in AMI diagnosis and is a valid source for future pharmacoepidemiological research in cardiovascular diseases.
